# Proinflammatory cytokines sensitise mesenchymal stromal cells to apoptosis

**DOI:** 10.1038/s41420-025-02412-0

**Published:** 2025-03-28

**Authors:** Natalie L. Payne, Swee Heng Milon Pang, Andrew J. Freeman, Dilara C. Ozkocak, Justin W. Limar, Georgia Wallis, Di Zheng, Senora Mendonca, Lorraine A. O’Reilly, Daniel H. D. Gray, Ivan K. H. Poon, Tracy S. P. Heng

**Affiliations:** 1 Department of Anatomy and Developmental Biology, Biomedicine Discovery Institute, Monash University, Clayton, VIC Australia; 2 Research Centre for Extracellular Vesicles, Department of Biochemistry and Chemistry, La Trobe Institute for Molecular Science, La Trobe University, Melbourne, VIC Australia; 3 The Walter and Eliza Hall Institute of Medical Research, Parkville, VIC Australia; 4 Department of Medical Biology, University of Melbourne, Parkville, VIC Australia; 5 Australian Research Council Training Centre for Cell and Tissue Engineering Technologies, Monash University, Clayton, VIC Australia

**Keywords:** Cell death and immune response, Acute inflammation

## Abstract

Mesenchymal stromal cells (MSCs) exert broad therapeutic effects across a range of inflammatory diseases. Their mechanism of action has largely been attributed to paracrine signalling, orchestrated by an array of factors produced by MSCs that are collectively termed the “secretome”. Strategies to enhance the release of these soluble factors by pre-exposure to inflammatory cytokines, a concept known as “licensing”, is thought to provide a means of enhancing MSC efficacy. Yet, recent evidence shows that intravenously infused MSCs entrapped within the lungs undergo apoptosis, and their subsequent clearance by host phagocytes is essential for their therapeutic efficacy. We therefore sought to clarify the mechanisms governing regulated cell death in MSCs and how exposure to inflammatory cytokines impacts this process. Our results show that MSCs are relatively resistant to cell death induced via the extrinsic pathway of apoptosis, as well as stimuli that induce necroptosis, a form of regulated inflammatory cell death. Instead, efficient killing of MSCs required triggering of the mitochondrial pathway of apoptosis, via inhibition of the pro-survival proteins MCL-1 and BCL-XL. Apoptotic bodies were readily released by MSCs during cell disassembly, a process that was inhibited in vitro and in vivo when the apoptotic effectors BAK and BAX were genetically deleted. Licensing of MSCs by pre-exposure to the inflammatory cytokines TNF and IFN-γ increased the sensitivity of MSCs to intrinsic apoptosis in vitro and accelerated their in vivo clearance by host cells within the lungs after intravenous infusion. Taken together, our study demonstrates that inflammatory “licensing” of MSCs facilitates cell death by increasing their sensitivity to triggers of the intrinsic pathway of apoptosis and accelerating the kinetics of apoptotic cell disassembly.

## Introduction

Mesenchymal stromal cells (MSCs) isolated from bone marrow (BM) and other tissues [1–3] exert immunomodulatory and anti-inflammatory effects that have the potential to be harnessed for therapeutic applications. Yet, despite 30 years of clinical investigations into MSC-based treatment for various diseases, including acute graft-versus-host disease (GvHD), Crohn’s fistula and acute respiratory distress syndrome in COVID-19 patients [4], most clinical trials failed to reach primary endpoints [5]. These outcomes highlight a gap in our knowledge of the mechanisms by which MSCs exert their therapeutic effects, which is important for identifying MSCs with therapeutic fitness and potency, as well as patient stratification approaches in future clinical trials, since only a proportion of patients may respond favourably to treatment with MSCs [6, 7].

The broad anti-inflammatory activity of MSCs in vitro has been attributed to their production of an array of soluble factors, collectively referred to as the MSC secretome. Exposure of MSCs to an inflammatory environment, which can to an extent be mimicked in vitro using various priming strategies [8], enhances the MSC secretome, a concept known as “licensing” [9, 10]. For example, human MSCs secrete IDO upon priming with IFNγ, which was shown to enhance their immunosuppressive potential in a mouse model of GvHD [11]. However, MSCs fail to engraft after intravenous administration [12, 13], challenging the concept that paracrine signalling of viable cells is solely responsible for in vivo MSC immunosuppression. Emerging evidence now indicates that the death of MSCs shortly after infusion may in fact be crucial to their therapeutic effects [14]. MSC apoptosis and consequent efferocytosis by phagocytes, observed in pre-clinical models of GvHD and allergic asthma [12, 15, 16], culminates in the secretion of anti-inflammatory mediators by monocytes and macrophages [12, 16–18] leading to therapeutic outcomes. This was further confirmed by the demonstration that GvHD and allergic asthma could be alleviated by direct administration of apoptotic MSCs [16, 19]. Similarly, apoptotic bodies and apoptotic vesicles released as a consequence of MSC apoptosis and cellular disassembly, were shown to promote cutaneous wound healing and alleviate type 2 diabetes through the polarization of macrophages towards an anti-inflammatory phenotype [20, 21]. Thus, the rapid apoptosis of MSCs in vivo may explain the paradox of MSC immunosuppression in the absence of engraftment.

Given the inflammatory microenvironment usually present in disease states can influence MSC fate and function [22, 23], unanswered questions include how MSCs are killed in different settings and how these mechanisms relate to their anti-inflammatory properties. MSCs can activate the complement and coagulation cascades, triggering their cell death upon contact with blood [24, 25]. In murine GvHD, MSC immunosuppression required host CD8^+^ T cell and CD56^+^ natural killer cell mediated MSC apoptosis through perforin-dependent cytotoxicity [16]. In immunocompromised mouse models, MSC apoptosis could still occur in the lungs despite the absence of host cytotoxic or alloreactive cells [12]. Of note, deletion of the mitochondrial apoptotic effectors, BAX and BAK, in MSCs prevented their apoptosis and abrogated MSC-mediated immunosuppression in models of allergic asthma and experimental autoimmune encephalitis [12]. These findings indicate that MSCs must undergo apoptosis as part of their in vivo immunosuppressive mechanism. Importantly, the induction of MSC apoptosis by patient-derived peripheral blood mononuclear cells (PBMCs) and the release of prostaglandin E2 by apoptotic MSCs was recently found to correlate with the clinical response in a cohort of fistulizing Crohn’s disease patients that received MSC therapy [26]. This study, linking MSC apoptosis to treatment efficacy, highlights the clinical relevance of understanding MSC death as a mechanism of immunosuppression.

Although cell death is a fundamentally conserved process, individual cell types vary in their susceptibility to the different regulated cell death pathways; tightly controlled signalling pathways that are distinct in terms of their initiation, execution and the nature of the resulting immune response. As many of the stimuli reported to license the immunosuppressive properties of viable MSCs (e.g., TNF, IFN-γ and toll-like receptor activation) can also induce death in other cell types [27–29], we aimed to probe the cell death and survival requirements of MSCs and how inflammatory licensing impacts this process. We found that apoptosis was efficiently triggered in MSCs by inhibiting pro-survival BCL-2 family proteins, but not by FAS ligation or necroptotic stimuli. We further identified that licensing of human MSCs with TNF and IFN-γ increases their sensitivity to BAX/BAK-executed mitochondrial apoptosis, providing a mechanism connecting this process to in vivo efficacy, with implications for MSC therapy.

## Results

### MSCs are resistant to the extrinsic pathway of apoptosis and necroptosis

**Fig. 1:**
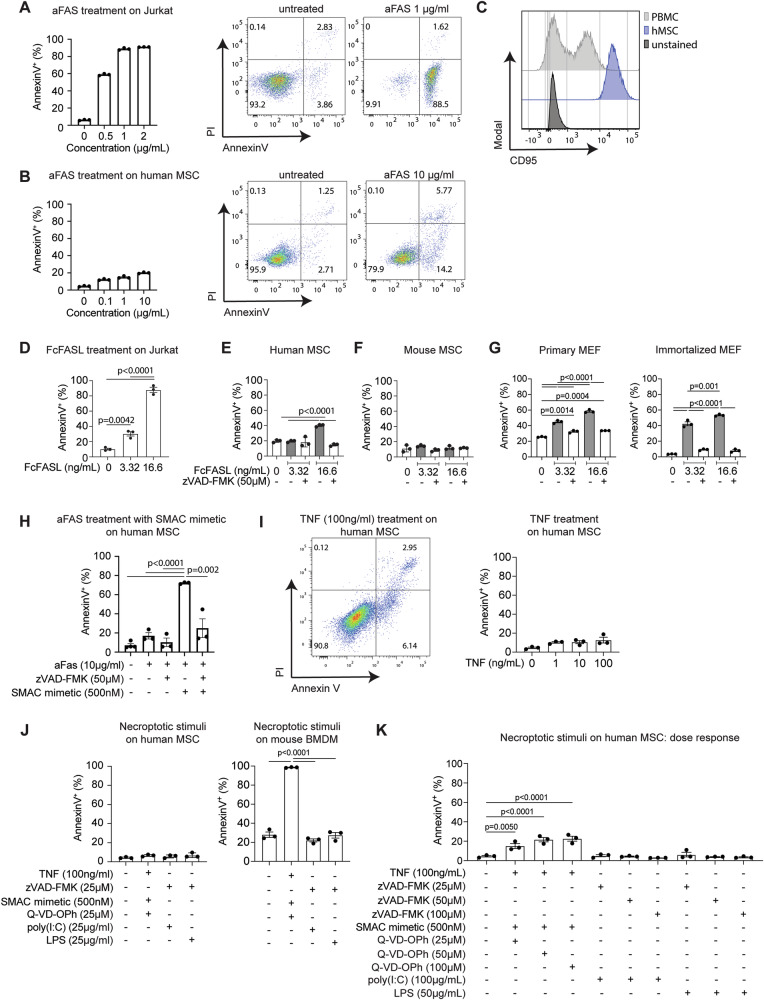
Human MSCs are not killed efficiently via the extrinsic pathway of apoptosis or necroptosis. **A** Quantification of Annexin V^+^ cells in Jurkat T lymphoma cells treated with increasing concentrations of anti-FAS antibody (aFAS) for 24 h (*n* = 3) and representative Annexin V/PI staining following stimulation with 1 μg/ml of anti-Fas antibody for 24 h. **B** Quantification of Annexin V^+^ cells in human BM-MSCs treated with increasing concentrations of anti-FAS antibody for 24 h (*n* = 3) and representative Annexin V/PI staining following stimulation with 10 μg/ml of anti-Fas antibody for 24 h. **C** Representative flow cytometric analysis of FAS expression in one of three human BM-MSCs in comparison to peripheral blood mononuclear cells (PBMC). **D** Quantification of Annexin V^+^ cells in Jurkat cells treated with increasing concentrations of FcFASL for 24 h (*n* = 3). **E**–**G** Quantification of Annexin V^+^ cells in human BM-MSCs (**E**), mouse BM-MSCs (**F**), and primary MEFs and SV40-immortalised MEFs (**G**) treated with increasing concentrations of FcFASL for 24 h with or without pre-treatment with 50 μM zVAD-FMK for 30 min (*n* = 3). **H** Quantification of Annexin V^+^ cells in human BM-MSCs treated with 10 μg/ml anti-FAS antibody for 24 h in the presence or absence of 500 nM SMAC mimetic (Compound A) with or without pre-treatment with 50 μM zVAD-FMK for 30 min (*n* = 3). **I** Representative Annexin V/PI staining in human BM-MSCs treated with 100 ng/ml TNF for 24 h, and quantification of Annexin V^+^ cells in human BM-MSCs treated with increasing concentrations of TNF (*n* = 3). **J** Quantification of Annexin V^+^ cells in human BM-MSCs (left panel) and mouse BMDMs (right panel) treated with 100 ng/ml TNF, or 25 μg/ml poly(I:C), or 25 μg/ml LPS for 24 h in the presence or absence of 500 nM SMAC mimetic, with or without pre-treatment with the pan-caspase inhibitors Q-VD-OPh or zVAD-FMK for 30 min (*n* = 3). **K** Quantification of Annexin V^+^ cells in human BM-MSCs treated with 100 ng/ml TNF, or 100 μg/ml poly(I:C), or 50 μg/ml LPS for 24 h in the presence or absence of 500 nM SMAC mimetic with or without pre-treatment with increasing concentrations of Q-VD-OPh or zVAD-FMK for 30 min (*n* = 3). Data expressed as the mean ± S.E.M. and representative of at least two independent experiments, *p* values by one-way ANOVA with Tukey’s post hoc test.

There are conflicting reports of MSC sensitivity to stimuli that trigger apoptosis via the extrinsic pathway [27, 28, 30, 31], a caspase-dependent form of regulated cell death resulting from extracellular perturbations detected by plasma membrane receptors [32]. We therefore first assayed the survival of human BM-derived MSCs (BM-MSCs) following FAS receptor ligation with an agonistic antibody against FAS. Human Jurkat T lymphoma cells, used as a positive control, were approximately 90% Annexin V^+^ following 24 h treatment with 1 µg/mL anti-FAS antibody (Fig. 1A). By contrast, human BM-MSCs exhibited little apoptotic cell death with increasing concentrations of anti-FAS antibody (Fig. 1B). Only approximately 20% apoptotic cells (Annexin V^+^PI^−^ and Annexin V^+^PI^+^) were observed at 10 µg/mL, despite the fact that BM-MSCs expressed high levels of FAS (Fig. 1C). Ligation of FAS with a different reagent, recombinant FcFASL (a trimeric form of FASL) [33], efficiently killed Jurkat cells (Fig. 1D), but was similarly ineffective in BM-MSCs, inducing apoptosis in ~40% of cells (Fig. 1E). Likewise, mouse BM-MSCs (Fig. 1F) also displayed increased resistance to FAS ligation compared to primary mouse embryonic fibroblasts (MEFs) and MEFs immortalised with SV40 (Fig. 1G).

To determine why MSCs were relatively resistant to death receptor-mediated apoptosis, we examined whether antagonising inhibitor of apoptosis proteins (IAP) could better activate caspase 8-dependent apoptosis. In the presence of the pan-IAP antagonist SMAC-mimetic, Compound A [34], human BM-MSCs exhibited robust cell death following treatment with anti-FAS antibody (Fig. 1H), suggesting that IAPs limit MSC sensitivity to FAS-mediated apoptosis. We confirmed that this apoptosis was caspase-dependent, as the effect was significantly abrogated in the presence of the broad spectrum caspase inhibitor, zVAD-FMK (Fig. 1H).

Next, we investigated the susceptibility of MSCs to necroptosis, an alternative, inflammatory form of cell death that can be initiated by certain TLR or TNF receptor signals when caspase-8 activity is inhibited [35]. Treatment with TNF alone for 24 h at concentrations up to 100 ng/ml did not induce significant cell death in human BM-MSCs (Fig. 1I). Moreover, the addition of the caspase inhibitor Q-VD-OPh and SMAC-mimetic failed to induce TNF-mediated necroptosis in human BM-MSCs, while close to 100% of mouse bone marrow-derived macrophages (BMDMs) were efficiently killed as previously reported [36] (Fig. 1J). Human BM-MSCs were also refractory to TLR-mediated necroptosis when stimulated with poly(I:C) or LPS in the presence of caspase inhibition with zVAD-FMK (Fig. 1J). The relative resistance of BM-MSCs to necroptotic stimuli was further confirmed by titration of caspase inhibitors up to 100 µM, which showed ~25% cell death induced by TNF-mediated necroptosis and negligible cell death in response to high doses of poly(I:C) or LPS (Fig. 1K).

Taken together, these data demonstrate that human MSCs are not killed efficiently via the extrinsic pathway of apoptosis, and are refractory to various necroptotic stimuli in the presence of caspase inhibition.

### Human and mouse MSCs rely on different BCL-2 family proteins for their survival

**Fig. 2:**
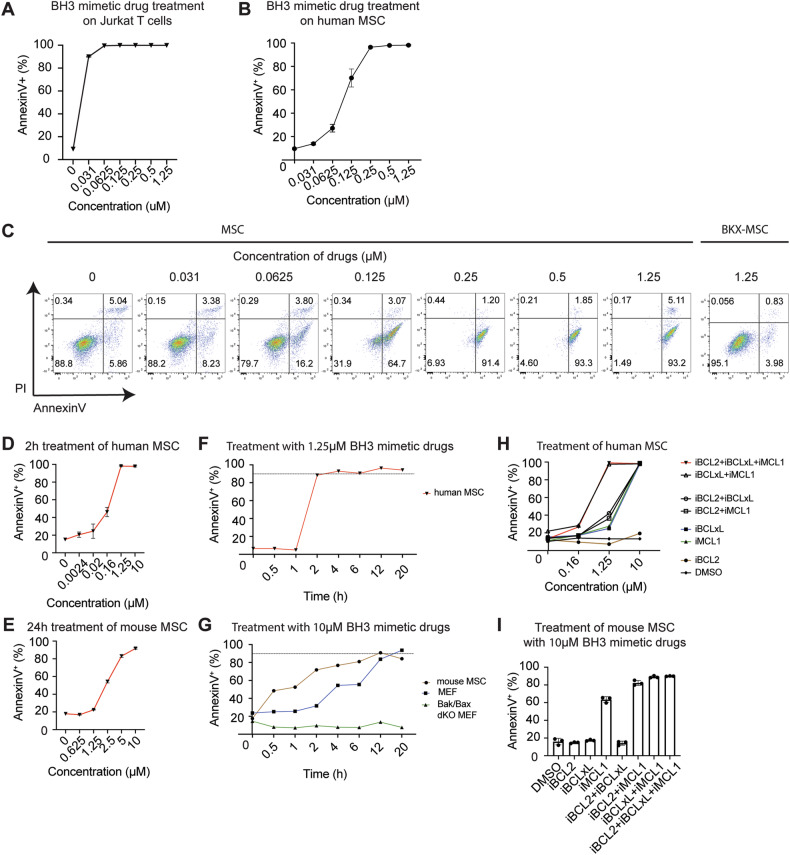
Efficient killing of human MSCs via the intrinsic pathway of apoptosis requires BCL-XL and MCL-1 inhibition. **A**, **B** Quantification of Annexin V^+^ cells in Jurkat cells (**A**) and human BM-MSCs (**B**) treated with increasing concentrations of the BH3 mimetic drugs ABT199 (BCL-2 inhibitor, iBCL2), A1331852 (BCL-XL inhibitor, iBCLxL) for 3 h. **C** Representative Annexin V/PI staining in human BM-MSCs treated with increasing concentrations of BH3 mimetic drugs, and BAK/BAX-deficient (BKX) MSCs treated with 1.25 μM BH3 mimetic drugs for 3 h. **D** Quantification of Annexin V^+^ cells in human MSCs (**D**) and mouse MSCs (**E**) treated with increasing concentrations of BH3 mimetic drugs for 2 h (*n* = 3). **F**, **G** Quantification of Annexin V^+^ cells at various time points following treatment of human MSCs with 1.25 μM BH3 mimetic drugs (**F**) and mouse MSCs, MEFs and BAK/BAX deficient MEFs treated with 10 μM BH3 mimetic drugs (**G**) (*n* = 3). **H** Quantification of Annexin V^+^ cells in human MSCs treated with increasing concentrations of various BH3 mimetic drugs combinations for 24 h (*n* = 3). **I** Quantification of Annexin V^+^ cells in mouse MSCs treated with various BH3 mimetic drugs combinations at 10 μM for 24 h (*n* = 3). Data expressed as the mean ± S.E.M. and representative of at least two independent experiments.

We next investigated the sensitivity of human MSCs to cell death via the intrinsic mitochondrial pathway of apoptosis, triggered by cellular stressors [32]. A class of small molecules termed BH3 mimetics specifically inhibit different pro-survival members of the BCL-2 family of proteins [37]. These include: ABT-199 (BCL-2 inhibitor [38]), A-1331852 (BCL-xL inhibitor [39]), and S63845 (MCL-1 inhibitor [40]). Using Jurkat cells as a positive control (Fig. 2A), human BM-MSCs were efficiently killed following treatment with the triple combination of these BH3-mimetic drugs in a dose-dependent manner, with maximal cell death induced after treatment for 3 h at 0.25 µM of each agent (Fig. 2B). Titration of the triple combination of BH3 mimetics demonstrated that the majority of cells exhibited an AnnexinV^+^PI^−^ early apoptotic phenotype within 2 h of 0.125 µM (Fig. 2C). By contrast, BKX-MSCs that do not express the intrinsic apoptotic effector molecules, BAX and BAK, as previously reported [12], remained viable at the highest dose tested (1.25 µM) (Fig. 2C, right). Reducing the treatment time to 2 h, human BM-MSCs were nearly 100% Annexin V^+^ at a concentration of 1.25 µM (Fig. 2D). In contrast, mouse BM-MSCs required a substantially longer treatment duration as well as a higher concentration of the BH3-mimetic drugs (10 µM) to achieve the same effect (Fig. 2E). This was confirmed over a time-course, whereby human BM-MSCs were Annexin V^−^ at 1 h, but approximately 90% Annexin V^+^ at 2 h when treated with1.25 µM BH3-mimetic drugs (Fig. 2F). Treatment of mouse BM-MSCs with 10 µM BH3-mimetic drugs resulted in a noticeably slower rate of killing, requiring 12 h to achieve over 90% cell death (Fig. 2G). Compared to mouse BM-MSCs, MEFs exhibited an even slower rate of killing, whereas MEFs deficient in BAX and BAK were resistant to death, as expected (Fig. 2G).

We next determined which pro-survival proteins were required for the survival of human or mouse MSCs. As expected, concurrent inhibition of BCL-2, BCL-XL, and MCL-1 using 1.25 µM of the BH3-mimetic drugs resulted in the majority of human BM-MSCs being killed. The same effect was observed when BCL-XL and MCL-1 were simultaneously inhibited (Fig. 2H), suggesting that human MSCs require combined inhibition of BCL-XL and MCL-1, but not BCL-2, to efficiently induce apoptosis via the intrinsic pathway. BCL-2 inhibition in combination with either BCL-XL or MCL-1 inhibition at 1.25 µM resulted in approximately 40% of cell death of human BM-MSCs, whereas sole inhibition of either BCL-XL or MCL-1 resulted in approximately 25% of cell death (Fig. 2H). At 10 µM, all combinations were able to efficiently induce cell death in human BM-MSCs, with the exception of BCL-2 inhibition alone (Fig. 2H). On the other hand, for mouse BM-MSCs, inhibition of only MCL-1 resulted in approximately 60% apoptosis (Fig. 2I), demonstrating that MCL-1 is the key pro-survival protein in mouse MSCs. MCL-1 inhibition in combination with either BCL-2 inhibition or BCL-xL inhibition resulted in over 80% of cell death of mouse BM-MSCs (Fig. 2I). These data reveal that BCL-2 is dispensable for MSC survival and that human MSCs are safeguarded by BCL-XL and MCL-1, whereas mouse MSCs predominantly require MCL-1 for cell survival.

### Human MSCs from different tissues have varying sensitivity to BH3-mimetic drugs

**Fig. 3:**
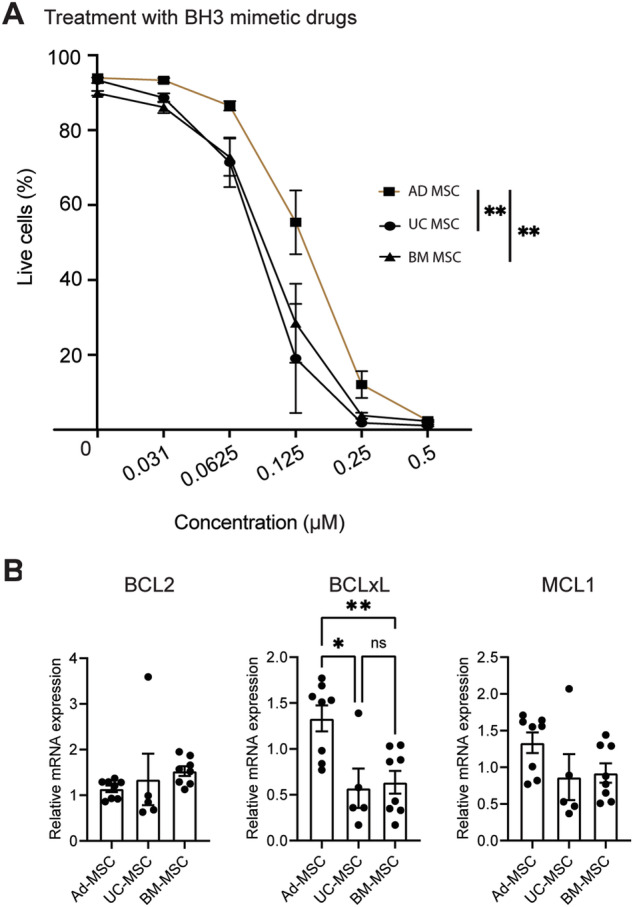
Adipose MSCs are less sensitive to killing via the intrinsic pathway of apoptosis. **A** Quantification of live cells (Annexin V^−^) in human AD-MSCs, UC-MSCs, and BM-MSCs treated with increasing concentrations of BH3 mimetic drugs for 2 h (*n* = 3 donors per tissue type). **B** Relative expression level of the anti-apoptotic genes *BCL-XL*, *BCL-2*, and *MCL-1* in cultured AD-MSCs, UC-MSCs, and BM-MSCs (*n* = 3 donors per tissue type). Data expressed as the mean ± S.E.M. pooled from two independent experiments, *p* values by one-way ANOVA with Tukey’s post hoc test.

MSCs derived from different tissues sources display heterogeneity in terms of their transcriptome, secretome and functional properties, including differential potential and immunomodulatory capacity [41]. We therefore compared the sensitivity of human MSCs derived from three common tissue sources to the BH3-mimetic drugs of interest. Human MSCs derived from adipose tissue (AD-MSC) treated with increasing concentrations of BH3-mimetics exhibited greater resistance compared to MSCs derived from umbilical cord (UC-MSC) and BM-MSCs (Fig. 3A). This finding was consistent across the three different AD-MSC donor lines tested. Human UC-MSCs and BM-MSCs exhibited a similar level of sensitivity to the BH3-mimetic drugs (Fig. 3A).

Steady-state quantitative PCR revealed that the relative transcription of *BCL2* was similar across different donors and tissue types (Fig. 3B, left). Although transcription of *BCL2A1* (encoding BCL-XL) (Fig. 3B, middle) and *MCL1* (Fig. 3B, right) was more variable, AD-MSCs expressed *BCL-XL* at significantly higher levels compared to UC-MSCs and BM-MSCs. Together, these data demonstrate that MSCs derived from adipose tissue exhibit a greater resistance to apoptosis induced by BH3 mimetics compared to MSCs derived from umbilical cord and BM, likely due to expression of higher amounts of these pro-survival molecules.

### Priming of MSCs by pro-inflammatory cytokines increases their sensitivity to apoptosis

**Fig. 4:**
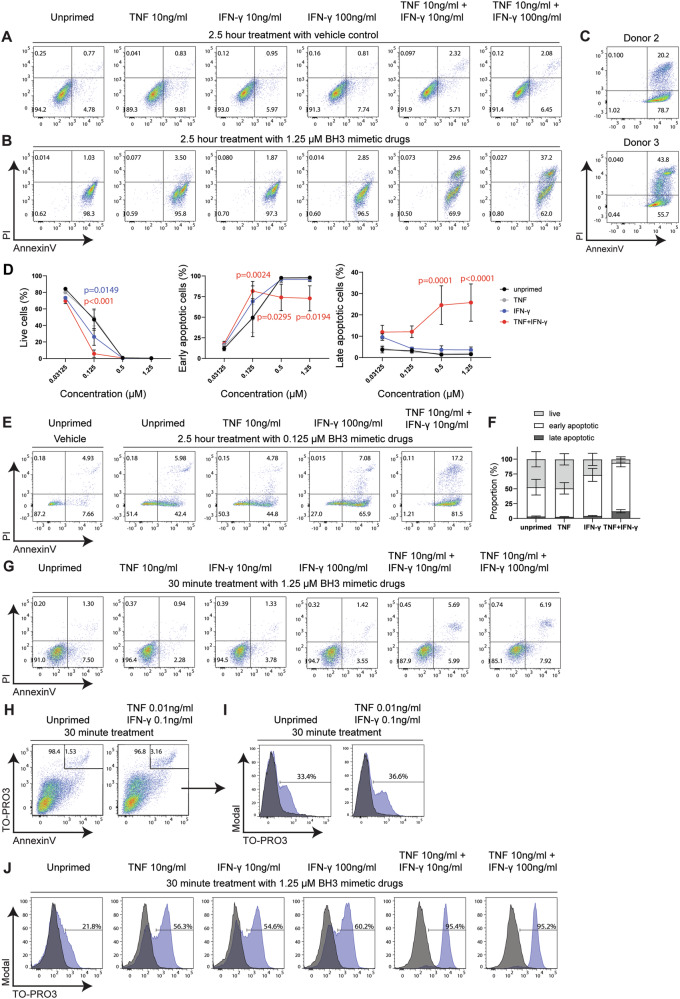
Inflammatory priming renders human MSCs more sensitive to the intrinsic pathway of apoptosis. **A**, **B** Representative Annexin V/PI staining in unprimed BM-MSCs and BM-MSCs primed with single (TNF or IFN-γ) or dual (TNF and IFN-γ) cytokines at the indicated concentrations for 24 h prior to treatment with vehicle control (DMSO) (**A**) or 1.25 μM BH3 mimetic drugs (**B**) for 2.5 h. **C** Representative Annexin V/PI staining of two additional BM-MSC donors primed with 10 ng/ml TNF and 100 ng/ml IFN-γ for 24 h prior to treatment with BH3 mimetic drugs for 2.5 h. **D** Quantification of the proportion of live Annexin V^−^PI^−^ cells (left panel), early Annexin V^+^PI^−^ (middle panel) and late Annexin V^+^PI^+^ apoptotic cells (right panel) in unprimed and primed BM-MSCs from three donors treated with increasing concentrations of BH3 mimetic drugs for 2.5 h (*n* = 3). **E** Representative Annexin V/PI staining in unprimed and primed BM-MSCs treated with 0.125 μM BH3 mimetic drugs for 2.5 h. **F** Quantification of live (Annexin V^−^PI^−^), early apoptotic (Annexin V^+^PI^-^) and late apoptotic (Annexin V^+^PI^+^) cells from three BM-MSC donors treated with 0.125 μM BH3 mimetic drugs (as shown in **E**) for 2.5 h (*n* = 3). **G** Representative Annexin V/PI staining in unprimed and primed MSCs treated with 1.25 μM BH3 mimetic drugs for 30 min. **H** Representative Annexin V/TO-PRO-3 staining and gating of Annexin V^+^TO-PRO-3^hi^ late apoptotic cells in unprimed and primed BM-MSCs treated with BH3 mimetic drugs for 30 min. **I**, **J** Representative histograms showing the proportion of TO-PRO-3^int^ cells after exclusion of Annexin V^+^TO-PRO-3^hi^ late apoptotic (as shown in **H**) in unprimed and primed BM-MSCs treated with BH3 mimetic drugs (blue histograms) or vehicle (grey histograms) for 30 min. Data expressed as the mean ± S.E.M. and representative of two independent experiments, *p* values by two-way ANOVA with Dunnett’s multiple comparison test.

Inflammatory licensing of MSCs is being employed as a strategy to improve MSC function and efficacy [14]. Priming cultured MSCs with cytokines such as IFN-γ, TNF and IL-1ß prior to infusion into patients is thought to mimic the inflammatory cues present at sites of tissue injury, which are required to induce the anti-inflammatory program in MSCs [9]. To determine whether inflammatory priming influences the sensitivity of MSCs to induction of apoptosis, we cultured human BM-MSCs with a combination of TNF and IFN-γ for 24 h prior to treatment with 1.25 µM BH3-mimetic drugs for 2.5 h. Exposure to TNF and IFN-γ alone without BH3 mimetic treatment did not trigger apoptosis, as previously reported in mouse MSCs [42, 43], since primed vehicle-treated human BM-MSCs remained AnnexinV^−^PI^−^ (Fig. 4A). While the proportion of AnnexinV^+^PI^−^ early apoptotic cells was unchanged when BM-MSCs were exposed to either TNF or IFN-γ alone, the combination of 10 ng/ml TNF and 10 ng/ml IFN-γ resulted in approximately 30% of BM-MSCs displaying an AnnexinV^+^PI^+^ late apoptotic cell phenotype (Fig. 4B). Priming with a tenfold higher dose of IFN-γ (100 ng/ml IFN-γ) increased the proportion of AnnexinV^+^PI^+^ late apoptotic BM-MSCs (Fig. 4B). We confirmed this finding with BM-MSCs from two additional donors. Up to ~40% of BM-MSCs primed with 10 ng/ml TNF and 100 ng/ml IFN-γ prior to apoptosis induction displayed an AnnexinV^+^PI^+^ late apoptotic phenotype (Fig. 4C). These results suggest that priming with inflammatory cytokines sensitises MSCs to the intrinsic pathway of apoptosis.

To further test whether priming increases MSC sensitivity to apoptosis, we treated BM-MSCs from all three donors with lower concentrations of BH3 mimetic drugs and examined the changes in their Annexin V and PI staining profile (Fig. 4D–F). At the lowest doses tested (0.03125 µM and 0.125 µM), the proportion of live (Annexin^−^PI^−^) cells was comparable between unprimed and TNF-primed BM-MSCs, but was significantly reduced by priming with IFN-γ or the combination of TNF and IFN-γ (Fig. 4D, left panel). This effect was most notable at 0.125 µM, where the proportion of live cells was reduced from approximately 50% in unprimed BM-MSCs to 25% in BM-MSCs primed with IFN-γ. TNF acted synergistically with IFN-γ to further reduce the proportion of live BM-MSCs to less than 1–2% (Fig. 4D, left panel, and Fig. 4E, F). At higher concentrations of BH3 mimetic drugs (0.5 µM and over), greater than 98% of unprimed BM-MSCs displayed an early Annexin V^+^PI^−^ phenotype, while approximately 25% of TNF and IFN-γ-primed BM-MSCs exhibited an Annexin V^+^PI^+^ late apoptotic profile (Fig. 4D, right panel). Overall these results confirm that inflammatory priming renders MSCs more sensitive to induction of apoptosis via the intrinsic pathway.

We next sought to examine how inflammatory priming influences the kinetics of apoptosis induction. At 30 min post BH3 mimetic drug treatment, BM-MSCs that were either unprimed, or primed with a single cytokine, remained Annexin V^−^PI^−^ (Fig. 4G). Only a small proportion (5–7%) of BM-MSCs primed with the dual combination of TNF and IFN-γ were AnnexinV^+^PI^+^. Efferocytosis involves the release of “find-me” signals from apoptotic cells to attract phagocytes prior to expression of “eat-me” signals, such as phosphatidylserine, that mediate engulfment [44]. To better resolve these early changes in apoptotic MSCs, we therefore examined activation of the plasma membrane channel, Pannexin 1 (PANX1). This channel is irreversibly activated during apoptosis due to cleavage at the C-terminus by caspase-3 and -7, triggering the release of “find-me” signals such as adenosine triphosphate (ATP) [45]. TO-PRO-3, a small monomeric nucleic acid stain, selectively enters cells during the early stages of apoptosis via the PANX1 channel, while the subsequent loss of cell membrane integrity during late apoptosis allows TO-PRO-3 to enter cells in a PANX1-independent manner [46]. We therefore used TO-PRO-3 to monitor early cell death progression in BM-MSCs by first gating out TO-PRO-3^hi^ late apoptotic cells (Fig. 4G), and then analysing the proportion of cells with intermediate TO-PRO-3 staining as an indicator of PANX1 activation. We identified that dual priming with low doses of TNF and IFN-γ (0.01 ng/ml and 0.1 ng/ml, respectively) prior to BH3 mimetic drug treatment did not initiate earlier activation of PANX1 channels, as the TO-PRO-3 staining profile between unprimed and primed BM-MSCs was comparable (Fig. 4H). Higher concentrations of TNF or IFN-γ alone (≥ 1 ng/ml), however, increased the proportion of BM-MSCs with activated PANX1 (Fig. 4I and data not shown). At 10 ng/ml, over 50% of BM-MSCs primed with either TNF or IFN-γ alone were TO-PRO-3^int^ compared to approximately 20% for unprimed BM-MSCs, while the majority (over 95%) of BM-MSCs were already TO-PRO-3^int^ after 30 min when primed with both cytokines (Fig. 4I, blue histograms). Importantly, primed BM-MSCs treated with DMSO (vehicle) only did not display this TO-PRO-3^int^ staining profile (Fig. 4I, grey histograms), demonstrating that inflammatory cytokines themselves do not activate PANX1 channels in MSCs. Taken together, these data show that inflammatory priming increases the sensitivity of MSCs to apoptosis.

### Inhibition of human MSC apoptosis reduces the release of apoptotic bodies in vivo

**Fig. 5:**
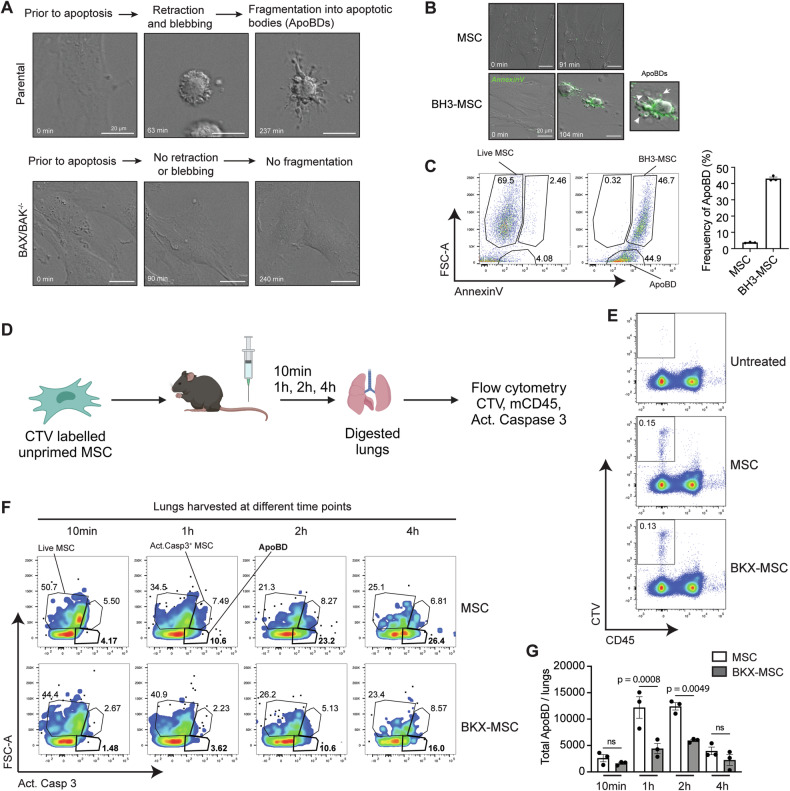
The intrinsic pathway of apoptosis is required for the release of apoptotic bodies in vivo*.* **A** Live cell imaging of parental MSCs (top panels; Video S1) and apoptosis-deficient BKX-MSCs (bottom panels; Video S2) following apoptosis induction with BH3 mimetic drugs. **B** Live cell imaging of untreated human bone marrow MSCs (top panels; Video S3) and BH3 mimetic drug-treated MSCs (bottom panels; Video S4) stained with Annexin V, showing formation of apoptotic bodies (arrows). **C** Representative Annexin V staining (left panel) and quantification of the proportion of apoptotic bodies (right panel) in human MSCs treated with BH3 mimetic drugs (*n* = 3). Data are expressed as the mean ± S.E.M and representative of three independent experiments. **D** Schematic for detection of MSCs and apoptotic bodies within the lungs at various time points after intravenous injection into mice. **E** Representative staining for CTV and CD45 to detect MSCs in digested lung tissue harvested 10 min post intravenous MSC injection **F** Representative staining for active caspase 3 within the CTV^+^ cell population, used to identify apoptotic MSCs and apoptotic bodies within digested lung tissue following intravenous injection of CTV-labelled parental MSCs (top panel) or BKX-MSCs (bottom panel). **G** Quantification of apoptotic bodies detected in the lungs of mice (as shown in **E**). Data represent the mean ± S.E.M. of *n* = 3 mice, *p* values by one-way ANOVA with Tukey’s post hoc test. Panel **D** was created with BioRender.com.

Following induction of apoptosis, cells undergo a coordinated disassembly process with distinct morphological changes. This includes plasma-membrane blebbing, membrane protrusion as well as fragmentation into subcellular fragments of 1–5 µM, termed apoptotic bodies [47]. Apoptotic body formation has predominantly been demonstrated in response to cell death stimuli in vitro. Using live cell imaging, we resolved BM-MSCs undergoing apoptotic cell disassembly following treatment with BH3 mimetic drugs, tracking the fragmentation and release of apoptotic bodies from Annexin V^+^ cells (Fig. 5A, B and Video S1–4). Further, we were also able to detect apoptotic bodies by flow cytometry, based on their relative size (FSC/SSC^lo^) and intermediate staining with Annexin V (Fig. 5C).

In vivo, mouse and human MSCs administered intravenously into BALB/c mice or immunodeficient mice rapidly undergo apoptosis within the lungs [12]. To evaluate apoptotic body formation by MSCs in vivo, lungs from mice injected with BM-MSCs were harvested over a time-course, digested and then activated caspase-3 was used as a marker of cells undergoing apoptosis (Fig. 5D). Within the CTV^+^CD45^-^ MSC population (Fig. 5E), apoptotic bodies could be identified by their small size and the presence of activated caspase-3 (Fig. 5F). Injection of apoptosis-resistant BKX-MSCs led to a marked reduction in apoptotic bodies detected ex vivo compared to the parental MSCs (Fig. 5F), confirming that they were derived from dying MSCs. Quantification of apoptotic bodies within the lungs revealed that amounts peaked at 1–2 h post injection for parental MSCs (Fig. 5G). They were significantly reduced in mice that received BKX-MSCs (Fig. 5G). These data confirm that intravenously injected MSCs release apoptotic bodies in vivo following entrapment within the lungs.

### Inflammatory priming accelerates the in vivo clearance of MSCs

**Fig. 6:**
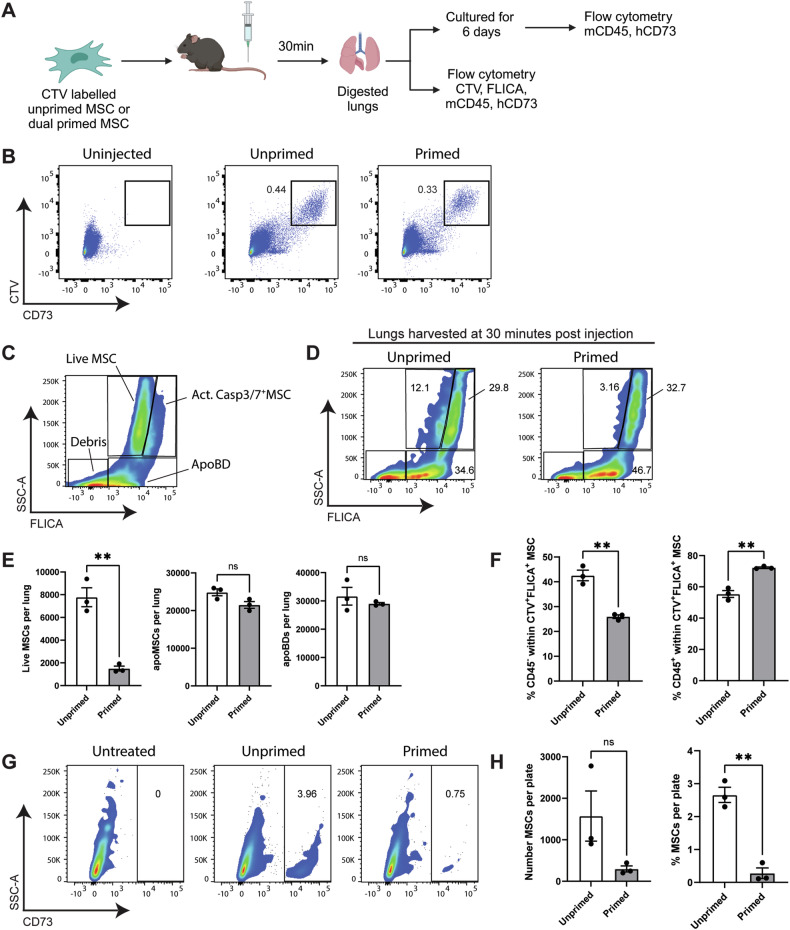
Inflammatory priming accelerates the clearance of apoptotic MSCs in vivo. **A** Schematic of how the survival of unprimed and primed MSCs was analysed within mouse lung tissue. **B** Representative staining for CTV and hCD73 to detect MSCs in digested lung tissue harvested 30 min post intravenous MSC injection. **C** Gating strategy used to identify live MSCs, apoptotic MSCs and apoptotic bodies, based on pooled samples of viable MSCs and BH3-mimetic drug treated MSCs stained with FLICA to detect active caspase 3/7. **D** Representative FLICA staining within the CTV^+^ cell population used to detect live MSCs, apoptotic MSCs and apoptotic bodies in digested lung tissue. **E** Quantification of the number of live MSCs, apoptotic MSCs and apoptotic bodies within the lungs (as shown in **D**). **F** Quantification of the proportion of CD45^−^ (left panel) and CD45^+^ cells (right panel) within the CTV^+^FLICA^+^ apoptotic MSC gate. **G**, **H** Detection of human CD73^+^ MSCs within ex vivo cultured lung cells. Digested lung cells from untreated mice, or mice that received intravenous unprimed MSCs or primed MSCs were cultured for six days and the number of human MSCs was quantified. **G** Representative staining of human CD73^+^ MSCs within the CD45^−^ population of cultured lung cells. **H** Quantification of the number (left panel) and proportion (right panel) of human CD73^+^ MSCs in cultured lung cells six days after plating. Data represent the mean ± S.E.M. of *n* = 3 mice, unpaired *T*-test; **p ≤ 0.01. Panel **A** was created with BioRender.com.

Next, we sought to determine how inflammatory priming impacted in vivo apoptosis of MSCs in the lungs. Unprimed or dual primed CTV-labelled BM-MSCs were administered to mice via intravenous injection and the apoptotic status of the injected MSCs was analysed within the lungs (Fig. 6A). At 30 min post injection, we could detect CTV^+^ CD73^+^ events within digested lung tissue from mice that received MSCs (Fig. 6B). The majority of CTV^+^ MSCs also stained positive for FLICA, indicative of activated caspase 3/7, and were identified as either apoptotic MSCs or apoptotic bodies (Fig. 6C, D). Only a small proportion of FLICA^−^ viable MSCs were detected. Overall, there was a significantly higher number of FLICA^−^ viable MSCs detected in the lungs of mice that received unprimed BM-MSCs compared to those mice that received primed BM-MSCs, but no differences in the number of apoptotic MSCs or apoptotic bodies (Fig. 6E). Furthermore, within the CTV^+^FLICA^+^ apoptotic MSC gate, a higher proportion of primed BM-MSCs were within the CD45^+^ population, likely indicating an interaction with or engulfment by host phagocytic cells (Fig. 6F).

To confirm that we were indeed detecting differences in the number of viable MSCs within the lungs, we re-plated cells from digested lung tissue as viable MSCs would adhere to tissue cultureware and propagate as colonies in culture. Analysis of human CD73 expression within the CD45^−^ population six days later (Fig. 6G) showed a significantly higher proportion of human CD73^+^ MSCs in cultures obtained from mice that had received unprimed BM-MSCs compared to those that received dual primed BM-MSCs (Fig. 6H). Together, these data support our in vitro findings that MSCs exposed to inflammatory cytokines are more sensitive to the intrinsic pathway of apoptosis, leading to accelerated in vivo clearance within the lungs.

## Discussion

Suboptimal and unpredictable outcomes in clinical trials has led to a re-evaluation of the mode of action of MSCs. Their short in vivo lifespan has been demonstrated using a variety of cell tracking methods, with the majority of intravenously administered MSCs passively entrapped and cleared from the lungs within 24 h post infusion [12, 18]. The ability of MSCs to suppress inflammatory responses at sites distal to the lungs, despite limited in vivo persistence, indicates that their mechanism of action does not rely on engraftment or soluble factors acting across long distances. Recent evidence suggests that this apparent paradox may be explained by MSC apoptosis and subsequent efferocytosis by host phagocytes engaging an immunosuppressive program that mediates therapeutic effects [12, 16, 26]. Understanding how MSCs die is therefore pivotal to unravelling their mechanisms of action and predicting patient clinical responses. Here, we examined the sensitivity of MSCs to different cell death stimuli and assessed how exposure to inflammatory cues impacts this process. Our data demonstrate that MSCs are most efficiently killed via the intrinsic pathway of apoptosis, and that their rapid apoptotic cell disassembly and in vivo clearance is accelerated by pre-exposure to inflammatory cytokines.

The functional response elicited from phagocytes upon clearance of dying cells is influenced by local environmental signals, the identity of the both the phagocyte and dying cell as well as the mechanism of cell death [48, 49]. For example, apoptotic cells release anti-inflammatory mediators [50] and engage different phagocytic receptors [49] to induce efferocytic programs that can promote resolution of inflammation or immunological tolerance. An immunogenic response, however, can be elicited if cells die via inflammatory forms of regulated cell death, such as necroptosis or pyroptosis, or due to reduced efferocytosis leading to secondary necrosis [48, 51]. To understand how dying MSCs interact with phagocytic cells to modulate immune responses, we sought to better define the cell death and survival mechanisms of MSCs. Our results showed the relative resistance of MSCs to cell death pathways triggered by ligands of death receptors and pattern recognition receptors. In contrast, robust and reproducible mitochondrial apoptosis could be induced in MSCs derived from different donors and tissue sources using BH3 mimetic drugs that target the pro-survival BCL-2 family proteins. Of note, we identified that MSCs treated with BH3 mimetic drugs in vitro or intravenously infused into mice readily produce apoptotic bodies during cell disassembly, a process inhibited when mitochondrial apoptosis was blocked due to combined loss of BAX and BAK. These results, demonstrating an essential role for the intrinsic pathway in triggering MSC apoptosis, are consistent with our previous data [12] and the caspase-8 independent apoptotic cell death of MSCs observed in response to serum deprivation and hypoxia, as reported by Zhu et al. [52].

Mechanistically, we identified that induction of mitochondrial apoptosis in human MSCs specifically required inhibition of both BCLXL and MCL-1, whereas inhibition of MCL-1 only was sufficient for induction of apoptosis in mouse MSCs. The higher concentrations of the MCL-1 inhibitor required for induction of mouse MSCs is consistent with the known species-specific differences in binding affinity of this small molecule, which is lower for rodent MCL-1 compared to human MCL-1 [53]. The reduced sensitivity of AD-MSCs to BH3 mimetics correlated with their higher transcription of pro-survival genes, in particular BCL-XL. Other studies have similarly highlighted the importance of BCL-XL in regulating apoptosis in MSCs [54] and fibroblasts, which are being targeted with BCL-XL inhibitors for treatment of scleroderma [55].

Our results also identified that MSCs are only efficiently killed through FAS ligation when IAPs are inhibited. This suggests that MSCs, like hepatocytes and pancreatic β cells, are so-called type II cells, which require amplification of the executioner caspase activation cascade via caspase 8-mediated cleavage of BID and activation of the mitochondrial pathway for efficient FAS-mediated killing [56]. While FAS engagement in type I cells decreases XIAP levels, type II cells show increased levels of XIAP, which can directly inhibit executioner caspases and consequently attenuate FAS-mediated apoptosis [57]. The conflicting reports on whether MSCs die following FAS engagement might be attributed to changes in the ratio of effector caspases and XIAP levels [58], possibly resulting from differences in dose and treatment time [59] as well as MSC tissue sources [28, 31]. Culture conditions can also contribute to how MSCs respond to death ligands. For example, MSC sensitivity to FAS-mediated apoptosis is increased under hypoxic conditions [27] and can be regulated by how they attach to 2D surfaces [60]. To this end, it is important to note that MSCs are also sensitive to anoikis [61], whereby the loss of integrin-mediated anchorage to the extracellular matrix activates apoptosis via the intrinsic pathway.

IFN-γ and TNF can act synergistically to induce inflammatory cell death in a variety of cell types, contributing to disease pathology in inflammatory bowel disease, sepsis and SARS-CoV-2 infection [62, 63], as well as promote apoptosis of pancreatic β cells in Type 1 diabetes [64]. Previous studies have also documented that TNF and IFN-γ induce apoptosis in mouse MSCs, limiting their survival after subcutaneous injection [42, 43]. In contrast, exposure of human MSCs to an inflammatory microenvironment, commonly recapitulated in ex vivo cultured MSCs by priming with cytokines such as TNF and IFN-γ, licenses their anti-inflammatory program [9]. Here, our results revealed that such licensing, whilst not directly inducing cell death, renders MSCs cells more sensitive to mitochondrial apoptosis. This finding is in contrast to FAS-induced apoptosis, which was not affected by TNF priming in MSCs [28]. Inflammatory primed MSCs displayed earlier activation of the PANX1 channel, externalisation of PS and loss of membrane integrity upon inhibition of the pro-survival BCL2 family of proteins with BH3 mimetic drugs.

Differences in the molecular mechanisms by which TNF and IFN-γ mediate cell killing have been identified between pancreatic β cells in Type 1 diabetes [64] and intestinal epithelial cells in Crohn’s disease [62]. TNF and IFN-γ have also reported to induce differential transcriptional profiles in MSCs depending on whether they are used alone or in combination [65, 66]. In contrast, one study showed that the heterogeneity between unprimed MSCs from different donors was lost upon dual priming [66]. Our results, although conducted with a limited number of donor lines, indicated some variability in the sensitivity of MSCs isolated from different tissues and donors to both BH3 mimetic drug treatment and inflammatory priming. It will therefore be of importance to identify the molecular targets of TNF and IFN-γ, define how these regulate the balance between pro- and anti-apoptotic signals in downstream pathways and interrogate the extent to which tissue source, donor and manufacturing processes influence the MSC response. Such information will be of importance when identifying which MSC donors are suitable for clinical applications. This is especially relevant considering that the interaction between an MSC product and the patient’s immune cells, specifically the ability to induce MSC apoptosis, may provide a tool for predicting patient clinical responses [26].

Our in vivo data, showing significantly reduced numbers of viable MSCs within the lungs and increased interaction with host CD45^+^ host cells, supports the concept that inflammatory priming of MSCs accelerates their clearance in vivo. We pre-exposed human MSCs to TNF and IFN-γ prior to in vivo administration, since mouse TNF crossreacts with the human receptor but mouse IFN-γ does not [67, 68]. However, other common inflammatory mediators, such as IL-1β, or TLR ligands [14], or those relevant to specific pathological conditions [69], are also likely play an important role in regulating MSC fate and function in vivo. A limitation of our study is that we did not directly visualise cell death and efferocytosis of unprimed and primed MSCs. New genetic tools, such as the Caspase and pH Activated Reporter, Fluorescence ON (CharON) reporter [70], will undoubtedly be instrumental in the context of in vivo mouse studies [71], where tracking efferocytosis has proven challenging. Further, in vitro studies employing human phagocytes, which can also display differential responses depending on the disease context and factors such as patient age [72, 73], will also be critical to understanding the impact of inflammatory priming and the clinical relevance of apoptotic MSCs as a mechanism of immunosuppression.

In summary, we have shown that induction of MSC apoptosis is most efficient when targeting the mitochondrial pathway, requiring co-inhibition of two members of the BCL-2 family of proteins, BCL-XL and MCL-1. This cell death pathway is critical for apoptotic cell disassembly and the release of apoptotic bodies in vivo. Inflammatory licensing of MSCs with IFN-γ and TNF prior to exposure to triggers of intrinsic apoptosis accelerates cell death and in vivo clearance of MSCs. This new insight into how MSCs die will enable a greater understanding of their mechanism of action and inform future strategies for enhancing their therapeutic efficacy.

## Materials and methods

### Reagents

BH3 mimetics ABT199 (BCL-2 inhibitor, iBCL2), A1331852 (BCL-XL inhibitor, iBCLxL) and S63845 (MCL-1 inhibitor, iMCL1) were purchased from Chemgood. Caspase inhibitors Q-VD-OPh and zVAD-FMK, Poly(I:C), LPS, propidium iodide, staurosporine and DNase I were purchased from Sigma-Aldrich. Anti-Fas human activating antibody (cat # 05-201, clone CH11) was purchased from Merck. FcFasL protein was purified from FcFasL-transfected HEK-293 as described [33]. The cell line was provided by Pascal Schneider (University of Lausanne, Switzerland). Compound A was produced as described [34]. Recombinant mouse PDGF-bb, human TNF and human IFN-γ were purchased from Peprotech. CellTrace^TM^ Violet (CTV) and Vybrant FLICA Apoptosis Assay kits were purchased from ThermoFisher Scientific. The following antibodies were purchased BD Bioscience: Annexin V FITC (cat # 556420), biotin anti-mouse TER119 (cat # 553672), CD31 (cat # 553371, clone MEC 13.3), CD45 (cat # 553078, clone 30-F11) and B220 (cat # 553085, clone RA3-6B2), anti-human activate Capase-3 (cat # 550821, clone C92-605) and anti-human CD73 (cat # 550257, clone AD-2). Collagenase type I was purchased from Worthington.

### Animals

Female 7- to 9-week old C57BL/6 and BALB/c mice were obtained from Monash Animal Services and maintained under specific pathogen-free conditions at the Monash University Animal Research Laboratories. All animal experiments were conducted in accordance with the guidelines of the Australian Code of Practice for the Care and Use of Animals for Scientific Purposes and approved by the Monash University Animal Ethics committee (Protocol ID 26022).

### Cell culture

Human bone marrow-derived MSCs were purchased from Tulane Center for Gene Therapy. Human adipose-derived MSCs were either purchased from ScienCell or isolated from subcutaneous adipose tissue obtained from outpatient liposuction procedures (Monash human ethics approval #2007/1798; performed with informed patient consent). Umbilical cord Wharton’s jelly-derived MSCs were either purchased from ScienCell or isolated from scheduled healthy caesarean sections (Southern Health ethics approval #2008000257; performed with informed patient consent). The isolation, culture and characterisation of MSCs has been described previously [74, 75]. Cryopreserved cells were cultured in MSC media for 24 h before use. Passage 3–6 cells were used, with all experiments involving induction of cell death, inflammatory priming and in vivo transplantation of human MSCs performed with passage 4 cells. Human MSCs deficient in *BAK* and *BAX* have been described previously [12] and were used at passage P5.

For mouse MSCs isolation, BM plugs were flushed from the femur and tibia of female C57BL/6 mice and then subjected to digestion as follows. BM plugs were resuspended by briefly vortexing in 2 ml pre-warmed RPMI-1640 medium (Sigma-Aldrich) containing 1500 U/ml collagenase Type I, followed by incubation for 20 min at 37 °C with occasional vortexing. The cell suspension was passed through a 70 μM strainer and the undigested BM was subjected to an additional two rounds of digestion. Cells from the three rounds of digestion were pooled and plated in tissue culture flasks at 1 × 10^6^ cells/cm^2^ in a 37 °C, 10% CO_2_, with media changes every 72 h. After seven days, cells were detached with TrypLE, stained with biotinylated antibodies against TER119, CD45, CD31 and B220 followed by anti-biotin microbeads (Miltenyi Biotec) according to the manufacturer’s instructions. Cells were passed through an LS column (Miltenyi Biotec) on a QuadroMACS^TM^ magnetic separator and the negative fraction containing MSCs was collected and cultured at 2000 cells/cm^2^ in MSC media supplemented with 5 ng/ml PDGF-bb.

Human Jurkat T lymphoma cells (clone E6-1; ATCC) were maintained in RP10 media (RPMI-1640 medium supplemented with 10% (v/v) FCS, 100 U/ml penicillin and 100 mg/ml streptomycin and 2mM L-glutamine) at 1 × 10^5^–1 × 10^6^ cells/ml in a 37 °C, 5% CO_2_ humidified incubator. Primary MEFs from C57BL/6 mice, SV40-immortalised MEFs and MEFs deficient in *Bak* and *Bax* [76] were maintained in DMEM medium supplemented with 10% FCS, 100 U/ml penicillin and 100 mg/ml streptomycin and 2mM L-glutamine and passaged with TrypLE when they reached 80% confluence. All cells tested negative for Mycoplasma.

### Induction of cell death and analysis by flow cytometry

Jurkat cells were seeded in 24-well tissue culture plates at 5 × 10^4^ cells per well in a volume of 0.5 ml immediately prior to induction of cell death. Adherent MSCs and MEFs were seeded the day prior at 2.5 × 10^4^ cells per well. Cell death was induced by removal of culture medium and the addition of apoptotic (anti-FAS, FcFASL, TNF) or necroptotic (TNF, LPS, poly(I:C)) stimuli in the presence or absence of SMAC mimetic (Compound A) at the indicated concentrations for 24 h at 37 °C. In experiments involving inhibition of caspases, cells were pre-treated with pan caspase inhibitors zVAD-FMK or Q-VD-OPh at the indicated concentrations for 30 min prior to the addition of apoptotic or necroptotic stimuli. For cell death induced via the intrinsic pathway, BH3 mimetic drugs were added at various concentrations and incubated between 30 min and 24 h at 37 °C as indicated. For experiments involving inflammatory priming, unless otherwise indicated cells were incubated with 100 ng/ml IFN or 10 ng/ml TNF alone, or in combination, for 30 min or 24 h prior to induction of intrinsic apoptosis with BH3 mimetic drugs at the indicated concentrations. Cells were harvested by pooling the supernatant containing non-adherent cells with adherent cells detached with TrypLE. After washing in 1× Annexin V binding buffer (10 mM HEPES, pH7.4, 140 mM NaCl, 2.5 mM CaC_2_ in distilled water), cells were stained with Annexin V FITC (5 μl in a total volume of 100 μl) for 15 min at room temperature in the dark. PI (2 μg/ml) or TO-PRO-3 (1.25 μM) in a volume of 100ul was then added, cells were placed on ice and samples were acquired via a LSRFortessa X-20 cell analyser (BD Biosciences) with BD FACSDIVA (BD Biosciences v6.0) and analysed using FlowJo v10 software.

### Live imaging of apoptotic MSCs

Human MSCs were seeded at 1.5 × 10^4^ cells/well in 8-well Nunc™ Lab-Tek™ II Chamber Slide™ (Thermofisher) at least 24 h prior to imaging. The day of imaging, cells were washed gently with pre-warmed DPBS before adding 300 µL of BH3-mimetic drug cocktail made up in complete MSC media. Cells were imaged using the 63× objective for 4 h on Zeiss Spinning Disk Confocal Microscope at 37 °C, 5% CO_2_, with 5 × 5 tile regions were collected.

### Detection of MSCs and apoptotic bodies in the mouse lung

Human MSCs were labelled with 5 μM of CTV according to manufacturer’s protocol and staining was confirmed by flow cytometry. The labelled MSCs (1 × 10^6^ in 200 μl DPBS) were then administered intravenously into randomly selected BALB/c or C57BL/6 mice. The lungs were harvested for analysis at the indicated timepoints post MSC injection. Mice were euthanised by pentobarbitone overdose. Lungs were snipped into small fragments and digested for up to 1 h in lung digestion media (300 U/mL Collagenase type I (Worthington) and 50 U/mL DNAse I (Sigma-Aldrich) in RPMI-1640 in a 37 °C water bath with occasional agitation using a pipette. The digested lung samples were passed through a 70-micron cell strainer and centrifuged. The cell pellet was resuspended in red blood cell lysis buffer, washed, enumerated and resuspended in FACS buffer for subsequent flow cytometry analysis. Cell counting was performed using a Z2 Coulter Counter (Beckman Coulter). For experiments involving detection of activated caspase 3, single cell suspensions were fixed and permeabilized using the BD Cytofix/Cytoperm solution kit and then stained with active caspase-3 prior to flow cytometric analysis. For experiments involving detection of activated caspase 3/7, single cell suspensions were stained with the Vybrant FLICA apoptosis assay kit according to the manufacturer’s instruction prior to staining with antibodies against mouse CD45 and human CD73. Gating was guided by stained lungs samples from uninjected mice and pooled samples of viable and BH3 mimetic drug-treated apoptotic MSCs, and numbers were enumerated with counting beads. For experiments involving detection of MSCs in ex vivo lung cultures, 0.1×10^6^ cells from digested lung tissue were plated in 100 mm tissue culture plates and cultured for six days in RP10 media. Adherent cells were harvested with TrypLE, counted and stained with antibodies against mouse CD45 and human CD73 to quantify the proportion and number of human MSCs. Samples for flow cytometric analysis were acquired on a LSRFortessa analyser (BD Biosciences) with BD FACSDIVA (BD Biosciences v6.0) and analysed using FlowJo v10 software.

### Quantitative PCR

MSCs were seeded in 24 well plates at 5 × 10^4^ cells per well in MSC medium. The following day cells were detached with TrypLE, washed twice in DPBS and RNA isolated using the RNeasy Micro Kit (Qiagen) according to the manufacturer’s instructions. cDNA was synthesized from 0.5 ng total using the QuantiTect Reverse Transcription Kit (Qiagen). Quantitative PCR was performed using QuantiFast SYBR Green PCR Kit (Qiagen) on an Eppendorf Mastercycler ep (Eppendorf) with 0.1 μl cDNA. The PCR cycling protocol consisted of an initial hold for 2 min at 50 °C (UDG incubation), followed by 2 min at 95 °C for enzyme activation, and then 40 cycles of 95 °C for 15 s, 57 °C for 15 s and 68 °C for 20 s followed by melt curve analysis. All reactions were performed with three technical replicates. Relative transcripts were calculated by the 2^−∆∆^Ct method using *ACTB* and *GAPDH* as reference genes. Primers were: BCLxL 5′-CATGGCAGCAGTAAAGCAAG-3′, 5′-GAAGGAGAAAAAGGCCACAA-3′; BCL2 5′-GAACTGGGGGAGGATTGTGG-3′, 5′-CCGGTTCAGGTACTCAGTCA-3′; MCL1 5′-ATGCTTCGGAAACTGGACAT-3′, 5′-TCCTGATGCCACCTTCTAGG-3′; ACTB 5′-CTGGCCGGGACCTGACAGACTACC-3′, 5′-ATCGGAACCGCTCGTTGCCAATAG-3′; GAPDH 5′-AACAGCGACACCCACTCCTC-3′, 5′-CATACCAGGAAATGAGCTTGACAA-3′.

### Statistical analysis

All statistical analyses were conducted using GraphPad Prism v10 with alpha set to 0.05. The unpaired student’s *t* test was used for comparison between two groups, and one-way independent measure ANOVA followed by Tukey’s post hoc test or two-way ANOVA with Dunnett’s multiple comparison test was used for comparison between three or more groups. Data were represented as mean ± SEM unless otherwise stated. A *p* value of ≤ 0.05 was considered significant. Each experiment was performed with three biological replicates and two independent experimental replicates were performed unless otherwise specified in the figure legends. All data meet the assumptions of the tests and the variance was similar between the groups being statistically compared. For animal studies, no statistical method was used to determine sample size. Mice were randomly allocated to experimental groups and no mice were excluded from analysis.

## Supplementary information

Video S1

Video S2

Video S3

Video S4

Supplementary Information

## Data Availability

The data generated in this study are available within the article and its supplementary data files. All raw data are available upon request.
