# Supplementary material for: Proinflammatory cytokines sensitise mesenchymal stromal cells to apoptosis
Source: Cell Death Discov. 2025 Mar 28;11:121. doi: 10.1038/s41420-025-02412-0 (PMC11950399; doi:10.1038/s41420-025-02412-0)
Supplement: Supplementary file 5 — Supplementary Information [file 41420_2025_2412_MOESM5_ESM.docx]

**Supplementary Information**

**Video S1.** Live cell imaging of BH3 mimetic drug-treated parental MSCs over 4 h, corresponding to images in the top panels of Figure 5A.

**Video S2.** Live cell imaging of BH3 mimetic drug-treated apoptosis-deficient BKX-MSCs over 4 h, corresponding to images in the bottom panels of Figure 5A.

**Video S3.** Live cell imaging of untreated human bone marrow MSCs stained with Annexin V (green), corresponding to images in the top panels of Figure 5B.

**Video S4.** Live cell imaging of BH3 mimetic drug-treated human bone marrow MSCs stained with Annexin V (green), corresponding to images in the bottom panels of Figure 5B.
